# Conventional-Vincristine Sulfate vs. Modified Protocol of Vincristine Sulfate and L-Asparaginase in Canine Transmissible Venereal Tumor

**DOI:** 10.3389/fvets.2019.00300

**Published:** 2019-09-18

**Authors:** Chanokchon Setthawongsin, Patharakrit Teewasutrakul, Sirikachorn Tangkawattana, Somporn Techangamsuwan, Anudep Rungsipipat

**Affiliations:** ^1^Companion Animal Cancer Research Unit, Department of Veterinary Pathology, Faculty of Veterinary Science, Chulalongkorn University, Bangkok, Thailand; ^2^Oncology Clinic, Faculty of Veterinary Science, Small Animal Teaching Hospital, Chulalongkorn University, Bangkok, Thailand; ^3^Department of Veterinary Pathobiology, Faculty of Veterinary Medicine, Khon Kaen University, Khon Kaen, Thailand

**Keywords:** apoptosis, canine transmissible venereal tumor (CTVT), chemotherapy, drug resistance, L-asparaginase, vincristine

## Abstract

**Background:** Vincristine (VCR) is a mono-chemotherapy for canine transmissible venereal tumor (CTVT). L-asparaginase (LAP) is usually used in combination with other drugs. Previously, LAP-VCR protocol was applied for the CTVT-VCR-resistant cases. However, there were a few reports about using this protocol since the first visit.

**Aims:** To firstly investigate the effectiveness of combining chemotherapy (Vincristine and L-asparaginase, VCR-LAP) in normal CTVT case. Secondly, to compare this protocol with the conventional (Vincristine, VCR) protocol before and during treatment in 24 CTVT dogs.

**Materials and Methods:** Clinical signs, tumor relative volume, and histopathological change [amount of CTVT cells, tumor-infiltrating lymphocytes (TILs), TILs/CTVT ratio, collagen area, and Ki-67 proliferative index (PI)] were the treatment evaluation parameters. Moreover, transcriptome analysis of apoptotic (*Bcl-2, Bax*), drug-resistant genes (*ABCB1, ABCG2*), and BCL-2 and BAX expression were also included.

**Results:** Both protocols gave the decreased tumor volume, increased TILs/CTVT ratios and collagen area in the mass. Interestingly, the combination protocol decreased treatment time. There were two resistant cases after treatment with VCR. The expression of *Bcl-2* and *Bax* were decreased, and this may indicate the better response after treatment. Moreover, both drug resistant genes did not increase after treatment.

**Conclusion:** The main finding of this study is that the combination protocol did not only decrease treatment duration time but also gave the effectiveness of treatment outcomes in CTVT cases. Therefore, the application of the new protocol could be used by the field practitioners.

## Introduction

Canine transmissible venereal tumor (CTVT) is known as an occurring allograft tumor (1). It usually occurs at the mucous membrane of external genital (GTVT) area (2, 3). In addition, CTVT mass also occurs at extragenital areas (ETVT) (4–8). As such, other round cell tumors should initially include in the differential diagnose (9). CTVT show a predictable growth pattern: first, the progression phase (P phase), second, the stable phase (S phase), and finally, the regression phase (R phase) (1, 3, 10–12).

Vincristine sulfate (VCR) is efficiently used as a mono-chemotherapeutic drug for CTVT worldwide. VCR has a specific effect in the cell cycle and induces tumor cells to apoptose (1). Although, the success of treatment by VCR is >90%, resistance to treatment can sometimes still occur. The average interval for treatment by VCR is ~4–8 applications or about 3–5 weeks (13–15). If regression is not achieved after 6 weeks of injection, such cases are classified as vincristine resistant cases (16). The major cause of drug resistance is due to multidrug-resistant (MDR) protein. P-glycoprotein (P-gp) and multidrug-resistant protein 1 (MDR1) are encoded by the *ABCB1* gene. Other important members include the multidrug resistance-associated protein 1 and 2 (MRP1 and MPR2) and breast cancer resistance protein (BCRP), which are encoded by *ABCC1, ABCC2*, and *ABCG2* genes, respectively. The drug resistance in canine multicentric lymphoma is associated with upregulation of *ABCB1* and *ABCG2* genes (17). These genes may be involved in efflux of vinca alkaloid drugs as well as doxorubicin, which is chemotherapeutic drug used for CTVT treatment. Previous studies reported that there was an existing modulator effect of vincristine on *MDR-1* gene expression. It was higher in CTVT cells and showed higher survival level after vincristine application (18). Moreover, CTVT Plasmacytoid cells showed higher P-gp expression and a potential drug resistance behavior (19).

Resistant VCR-treated CTVT cases can occur, and doxorubicin was the drug of choice as stated in those previous reports (13, 15). However, additional L-asparaginase (LAP) was applied to VCR-resistant cases in one study (16). LAP is an enzyme which inhibits the protein synthesis and induces tumor cell death. The advantage of using LAP was that all dogs treated with LAP did not show any adverse effects. Moreover, LAP is well-tolerated as an effective drug with a unique mechanism of action and is not involved with the *MDR-1* gene (20, 21). Nowadays, information about LAP application in resistant CTVT cases and normal CTVT cases is still limited.

Apoptosis of tumor cells can be induced by chemotherapeutic drugs. The intrinsic pathway is regulated by the Bcl-2 family. BAX is a pro-apoptotic protein which triggers the mitochondrial membrane permeability in response to apoptotic stimuli. Conversely, BCL-2 is an anti-apoptotic protein which protects cell death (22). Therefore, the balance between them is important (23, 24). In other studies, down-regulation and up-regulation of Bax and Bcl-2 expressions and Bax-Bcl-2 ratio were associated with malignancy or survival criteria prediction (25–29). In cancer research, Ki-67 expression is regarded as a marker for cellular proliferative rate. The detection of its expression is usually connected with a high frequency of metastasis and high malignancy (30, 31). However, the relationship among the apoptotic-related factors, proliferating factors and chemotherapeutic drugs is not fully understood in CTVT cases.

This study is aimed to (1) investigate the effectiveness of VCR-LAP in CTVT cases with modification of the dosage and route of administration of LAP. (2) Compare and investigate the effective chemotherapeutic protocol between VCR and VCR-LAP). (3) Determine the relative level of mRNA expression and protein expression of Bax and Bcl-2; Ki-67 proliferative index (PI) in CTVT tissues before and during treatment with 2 chemotherapeutic protocols.

## Materials and Methods

### Animals

The study design was randomized, double-blinded, and placebo controlled. All CTVT dogs were based on inclusion criteria: (1) complete blood count (CBC), and serum chemistry profile were in suitable range for chemotherapeutic treatment. Moreover, blood parasites were also examined by thin blood smears and SNAP 4Dx Plus Test kit (IDEXX, USA), (2) The dogs must have been previously diagnosed by cytology, histopathology and confirmed by polymerase chain reaction assay for definitive diagnosis (9). If the patients had abnormal blood results, chemotherapy was postponed until the adverse events were normal with only supportive treatment during the meantime. (3) Complete follow-up data including treatment recurrence, treatment side effects and the number of treatment application.

The CTVT mass type was anatomically classified into GTVT type for mass, located only in the genital area, ETVT type when the mass is located at other parts of the body within/without involvement of the genital area.

### Chemotherapeutic Protocol and Sample Collection

The CTVT dogs were randomly divided into 2 groups and the chosen chemotherapeutic was given accordingly. This was due to the fact that affected dogs did not come to the animal hospital at the same time or during the same period. We divided the animal into GTVT or ETVT and then acquired the ordering number, in order to give 2 chemotherapeutic protocols in the same proportions of each group. In the case of the treatment group, which did not come to follow our protocol, we were looking for the new case instead of previous case. In addition, there were two chemotherapeutic protocols which involved treating patients in this study: (1) conventional protocol: 0.025 mg/kg of Vincristine sulfate; VCR (VCS, Boryung Pharm) given IV weekly. (2) Modified combination protocol: combined 200 IU/kg or 5,000 IU/m^2^ L-asparaginase; LAP (Leunase, Hyowa Hakko Kirin), given SC at week 1 and 3, and 0.025 mg/kg VCR given IV at week 2 and 4 for at least 1 cycle (Table 1).

**Table 1:** Chemotherapeutic protocols: conventional protocol (vincristine, VCR) and modified combination protocol (vincristine and L-asparaginase, VCR-LAP).

**Chemotherapeutic drugs**	**Week of treatment**								
	**0**	**1**	**2**	**3**	**4**	**5**	**6**	**7**	**8**
**Conventional chemotherapeutic protocol**									
Vincristine sulfate 0.025 mg/kg (IV)		^*^	^*^	^*^	^*^	^*^	^*^	^*^	^*^
**Modified combination chemotherapeutic protocol**									
Vincristine sulfate 0.025 mg/kg (IV)			^*^		^*^		^*^		^*^
L-asparaginase 5,000 IU/m^2^ (SC)		^*^		^*^		^*^		^*^	

* *Week for given the drug*.

There were 11 dogs included in VCR (6 with GTVT and 5 with ETVT), and 13 dogs in VCR-LAP (7 with GTVT and 6 with ETVT). Each tumor mass was sampled every week at week 0, 1, and 2 or until no presented mass. Cytology, histopathology, and immunohistochemistry (IHC) were performed for further analysis. Moreover, additional fresh tissues were kept at −80°C for molecular assays. All sampling procedures were approved by the Animal Care and Use Committee (No. 13310077).

### Treatment Evaluation

#### Clinical Signs and Gross Lesions

Dogs from each protocol were physically examined weekly upon chemotherapeutic protocol. The pet owners were questioned regarding general signs and signs of gastrointestinal toxicities, and the hematologic toxicity was graded according to the criteria for adverse events following chemotherapy of veterinary cooperative oncology group (VCOG-CTCAE) (32), and recorded. Each GTVT case, tumor mass's width, length, and thickness were measured with Vernier caliper. The total width, length and thickness were summarized in condition with more than one mass in ETVT cases. The tumor volume was calculated by (¶ × width × length × thickness)/4 (33). In addition, the measurement with radiograph images was also used in the oro-nasal case. The tumor sizes were graded followed by distributing the tumor based on tumor response criteria (15). The TNM stage was evaluated according to the TNM-WHO classification of tumors in domestic animals (34). To examine the size of tumor, the tumor relative volume was calculated weekly as total tumor volume divided by body surface area and recorded for further analysis.

#### Cytomorphology Classification, Histopathology, and Immunohistochemistry

The cytologic slides were then fixed in absolute methanol, stained with Giemsa (Merck, Darmstadt, German), evaluated for diagnosis and classified according to the cytomorphologic classification criteria (35). The cytomorphologic type (plasmacytic, lymphocytic, or mixed type) was used as clinical information and used for evaluation of the correlation with effectiveness of the treatment and malignant behavior.

Tissue samples were fixed and had undergone routine histopathologic procedure (H&E staining). Each slide was examined by a veterinary pathologist, in order to evaluate the number of tumor cells, tumor infiltrating lymphocytes (TILs) and other pathological changes. TILs/tumor cells ratio was estimated by counting the numbers of tumor cells and TILs in 10 random high-power fields (HPF, 40x), and an average was calculated for each slide. Moreover, Masson's Trichrome Stain was also used to evaluate collagen production or fibrosis area.

To evaluate Ki-67 Proliferative Index (Ki-67 PI), the tissues were deparaffinized and rehydrated with a series of graded alcohol. The slides were pretreated for antigen retrieval with a microwave for 20 min, followed by applying Ki-67 antibody (Table 2) and were incubated overnight at 4°C. All slides were immunostained with polymer-based, non-avidin-biotin EnVision detection system^TM^ (Dako, Denmark), and 3, 3′-diaminobenzidine tetrahydrochloride (DAB) substrate (Dako, Denmark) as a visualization system. The Ki-67 PI was defined as the percentage of nuclear immunolabeling positive cells (at least 1,000 cells were counted) among the total number of tumor cells counted in at least 10 HPF (36).

**Table 2:** Primary antibodies, antigen retrieval solution, their dilutions, and positive area.

**Antibody**	**Source**	**Pretreated**	**Dilution**	**Positive area**
Ki-67 (MM1)	Leica, UK	Tris EDTA, pH9 Microwave, 20 min	1:100 overnight	Nucleus
Bcl-2	Leica, UK	Citrate, pH6 Autoclave, 121°C, 10 min	1:100 overnight	Membrane and cytoplasm
Bax	Dako, Denmark	Citrate, pH6 Autoclave, 121°C, 10 min	1:200 overnight	Cytoplasm

To study BCL-2 and BAX protein expression, IHC was performed on each sample. Briefly, the tissues were deparaffinized and rehydrated with a series of graded alcohol. Antibody panels were used as described in Table 2 with polymer-based non-avidin-biotin EnVision detection system™ (Dako, Glostrup, Denmark) and DAB substrate (Dako, Glostrup, Denmark), as the visualization system. The slides were then counterstained by Mayer's hematoxylin after immunolabeling color development (Table 2) (29, 37). The slides were incubated without primary antibodies and were included for each staining as a negative control. Canine lymphoma sections were used as positive control.

### Quantitative Image Analysis

The digital imaging system, comprising a Zeiss Primo Star microscope (Zeiss, Oberkochen, Germany) and a Canon EOS 550D camera (Canon, Tokyo, Japan), was used for image digitization. Ten HPFs were randomly captured per slide. I-solution program was used to quantify protein expression, BCL-2 and BAX immunolabeling protein expression was determined as the cancer area labeled intensity (μm^2^) in each slide, and an average was calculated. Moreover, Masson's Trichrome stained slide was also digitalized in 10 random HPFs. The blue staining areas were indicated as collagen formation area or fibrosis area and were measured by I-solution program (IMT, Canada). An average was calculated by Excel software (Microsoft, USA).

### Transcriptome Analysis of Apoptotic and Drug Resistance Gene: Quantitative Reverse Transcription Real -Time PCR (qRT-PCR)

Total RNA was extracted from CTVT samples (*n* = 24) using a Nucleospin RNA kit (Macherey-Nagel, Germany) according to the manufacturer's protocol. Briefly, the homogenized sample was lysed, and then the total lysate was trapped in a membrane column. The genomic DNA was removed by treating the RNA samples with RQ1 RNase-Free DNase (Promega, Madison, USA). Finally, total RNA was eluted and kept in a sterile tube. RNase-free, DNAse-treated RNA concentration, and purification were determined using NanoDrop Lite spectrophotometer (Thermo Fisher Scientific Inc., Wilmington, USA).

Five hundred nanogram of total RNA was used as a template to construct a cDNA with Omniscript^®^ Reverse Transcription Kit (Qiagen, German), according to the manufacturer's protocol. Rotor gene^®^ Q thermocycler (Qiagen, German) was performed with KAPA SYBR^®^ Fast qPCR Kit Master Mix (2X) universal for qRT-PCR, according to the manufacturer's protocol with the primers for housekeeping gene as β*-actin*, which showed the highest stability comparing hypoxanthine phosphoribosyltransferase *(HPRT)*, TATA box binding protein (*TBP)*, and glyceraldehydes-3-phosphate gehydrogenase (*GAPDH)* (Supplement data Table 4). The β*-actin* and interesting genes as *Bax, Bcl-2, ABCB1*, and *ABCG2* genes presented in Table 3 (17, 38–40). Briefly, 10 μL of 2X qPCR master mix and 200 nM of each primer mixed together with 20 ng of cDNA and adjusted to a final volume of reaction to 20 μL. qPCR reaction: firstly, the sample underwent enzyme activation at 95°C for 3 min, followed by 40 cycles of denaturation and annealing/extension at 95°C for 3 s and 60°C for 30 s, respectively. The threshold of fluorescence detection was set at the number of the threshold cycles (Ct), corresponding to the inflection point of the fluorescence curve from the baseline to the exponential phase. Each reaction was performed in triplicate intra-assay, and in duplicate inter-assay validations. A melting curve range from 75°C to 95°C was set to analyze the specificity of the PCR product. Real-time PCR efficiency ranged from 85 to 100%, which was obtained from standard curves of normal dog cDNA dilution series. In each sample, the Ct value with at least 1 duplicate Ct difference was used for analysis relative gene expression with the 2^−ΔΔ*ct*^ method (41).

**Table 3:** Primers used for real time polymerase chain reaction.

**Gene**	**Sequence**	**Product (bp)**	**References**
***Beta-actin***	5′-ATG GAA TCA TGC GGT ATC CAC-3′ 5′-CTT CTG CAT CCT GTC AGC AA-3′	141	(38)
***ABCB1***	5′-CAG TGG TTC AGG TGG CCC T-3′ 5′-CGA ACT GTA GAC AAA CGA TGA GCT-3′	79	(39)
***ABCG2***	5′-GGT ATC CAT AGC AAC TCT CCT CA-3′ 5′-GCA AAG CCG CAT AAC CAT-3′	143	(39)
***Bcl-2***	5′-CAT GCC AAG AGG GAA ACA CCA GAA-3′ 5′-GTG CTT TGC ATT CTT GGA TGA GGG-3′	76	(40)
***Bax***	5′-TTC CGA GTG GCA GCT GAG ATG TTT-3′ 5′-TGC TGG CAA AGT AGA AGA GGG CAA-3′	79	(40)

### Statistical Analysis

To evaluate the differentiation between the conventional and modified combination chemotherapeutic protocols, non-parametric Mann-Whitney *U*-test (2 independent samples) was used. A Friedman repeated measures analysis of variance and Wilcoxon Signed-Rank Test were used to determine the significance of differentiation in relative tumor volumes, TILs/CTVT ratio, collagen accumulation, Ki-67 PI, BCL-2, and BAX protein expression, as well as relative mRNA expression before (wk0) and during treatment (wk1 and wk2) in each group. The analysis was performed with the statistical pack SPSS program (IBM, USA) and a *p*-value of < 0.05 was considered as statistically significant. Data are also presented in graphs by GraphPad Prism program (GraphPad Software, USA).

## Results

### Clinical Information and Tumor Responsive Criteria

This prospective study during 2016 to 2017 was performed on 24 CTVT dogs including 13 males and 11 females, with ages ranging from 1 to 10 years old (mean 3.8 years old), and all of them were mixed breed dogs. In GTVT cases, most of them were in T3 stage, following the TNM staging criteria. In addition, the majority of the ETVT masses were located at subcutis. Moreover, the masses were also located at the nasal cavity, oral cavity and superficial lymph nodes (Tables 4, 5).

**Table 4:** Clinical information, cytomorphologic type, TNM staging, and response to treatment with VCR protocol.

**No**.	**Age (year)**	**Sex**	**Type**	**Area of lesion**	**Cytomorphologic type**	**TNM stage**	**Treatment time (responsive)**	**General sign/lethargy**	**Weight loss**	**GI toxicity**	**Hematologic toxicity**
1	2	F	GTVT	Vulva and vagina	Mixed	T3	2 weeks (CR)	G1	G1	G1	G0
2	4	F	GTVT	Vulva and vagina	Lymphocytic	T3	5 weeks (CR)	G0	G1	G1	G0
3	3	F	GTVT	Vulva and vagina	Plasmacytic	T3	6 weeks (CR)	G0	G1	G1	G0
4	4	F	GTVT	Vulva and vagina	Plasmacytic	T3	6 weeks (CR)	G0	G0	G0	G0
5	1	F	GTVT	Vulva and vagina	Plasmacytic	T3	5 weeks (CR)	G0	G1	G0	G0
6	1	F	GTVT	Vulva and vagina	Plasmacytic	T3	4 weeks (CR)	G0	G0	G0	G0
7	6	M	ETVT	Nasal cavity and oral cavity	Mixed	T3N1M	>8 weeks (resistance)	G0	G0	G2	G0
8	5	M	ETVT	Prepuce, skin, and conjunctiva	Plasmacytic	T2N1M	4 weeks (CR)	G0	G0	G0	G0
9	3	M	ETVT	Prepuce, inguinal LN, and penis	Plasmacytic	T3N1M	6 weeks (CR)	G0	G1	G2	G0
10	6	M	ETVT	Inguinal LN and prepuce	Mixed	T3N1	6 weeks (recurrent)	G0	G0	G0	G0
11	10	F	ETVT	Skin	Plasmacytic	T3	2 weeks (CR)	G0	G2	G0	G0

*F, female; M, male; GTVT, genital canine transmissible venereal tumor; ETVT, extragenital canine transmissible venereal tumor; LN, lymph node; T, tumor; N, lymph node; M, metastasis; CR, complete response; PR, partial response; G, grade*.

**Table 5:** Clinical information, cytomorphologic type, TNM staging, and response to treatment with VCR-LAP protocol.

**No**.	**Age (year)**	**Sex**	**Type**	**Area of lesion**	**Cytomorphologic type**	**TNM stage**	**Treatment time (responsive)**	**General sign/lethargy**	**Weight loss**	**GI toxicity**	**Hematologic toxicity**
1	3	M	GTVT	Penis	Plasmacytic	T3	3 weeks (CR)	G0	G1	G0	G0
2	2	M	GTVT	Penis	Plasmacytic	T2	4 weeks (CR)	G0	G1	G0	G0
3	2	F	GTVT	Vulva and vagina	Plasmacytic	T3	3 weeks (CR)	G0	G1	G0	G0
4	2	M	GTVT	Penis	Plasmacytic	T3	2 weeks (CR)	G0	G0	G0	G0
5	5	F	GTVT	Vulva and vagina	Plasmacytic	T3	2 weeks (CR)	G0	G0	G0	G0
6	1	F	GTVT	Vulva and vagina	Mixed	T2	3 weeks (CR)	G1	G0	G1	G0
7	4	M	GTVT	Penis	Plasmacytic	T3	4 weeks (CR)	G0	G0	G0	G0
8	9	M	ETVT	Skin	Plasmacytic	T3N1M	2 weeks (death)	G1	G3	G2	G0
9	5	M	ETVT	Skin and LN	Plasmacytic	T2N1M	2 weeks (CR)	G0	G0	G0	G0
10	3	F	ETVT	Skin, vulva, and vagina	Plasmacytic	T3N1M	3 weeks (CR)	G0	G1	G1	G0
11	4	M	ETVT	Skin, penis, and LN	Lymphocytic	T3N1M	2 weeks (CR)	G0	G1	G0	G0
12	4	M	ETVT	Skin	Plasmacytic	T3	5 weeks (CR)	G0	G0	G1	G0
13	3	M	ETVT	Skin and penis	Plasmacytic	T3N0M	5 weeks (CR)	G0	G1	G0	G0

*F, female; M, male; GTVT, genital canine transmissible venereal tumor; ETVT, extragenital canine transmissible venereal tumor; LN, lymph node; T, tumor; N, lymph node; M, metastasis; CR, complete response; PR, partial response; G, grade*.

**Figure 1:**
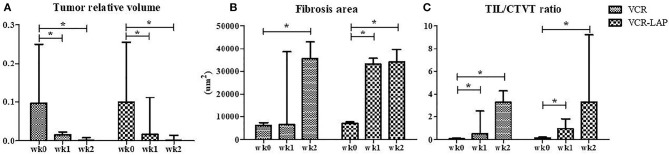
The tumor relative volume, fibrosis area and TIL/CTVT ratio of CTVT samples before treatment (wk0) and during treatment (wk1–2 PT). The tumor relative volume had a decreasing trend **(A)**. On the other hand, the areas of fibrosis had the increasing trend significantly **(B)**. The TIL/CTVT ratio had a trend of increasing due to the increasing of TILs and decreasing of CTVT cells **(C)** [Friedman repeated measures analysis of variance and Wilcox Signed-Rank test was used to determine the significance of differentiation before (wk0) and during treatment (wk1 and wk2) in each group, ^*^p < 0.05].

Before treatment, GTVT masses were cauliflower-like mass, fragile and oozed blood. On the other hand, ETVT masses showed more variable features depending on their locations. Gross lesions in both treated groups were improved as observed by the decrease in size, decrease in bleeding and decrease in attachment to the normal tissue (Supplement data Figure 1). The tumor volume had also gradually decreased after treatment. The relative tumor volume was compared between pre-treatment (wk0) and post treatment as wk1 post-treatment (wk1-PT) and wk2 post-treatment (wk2-PT). The tumor relative volume of pre-treated samples and treated samples in the same protocol was significantly different in both VCR (*p* = 0.003) and VCR-LAP (*p* = 0.001). The result revealed decreasing trends in both protocols. However, there was no statistical significance among the treatment protocols (Figure 1A and Supplement data Table 1).

**Figure 2:**
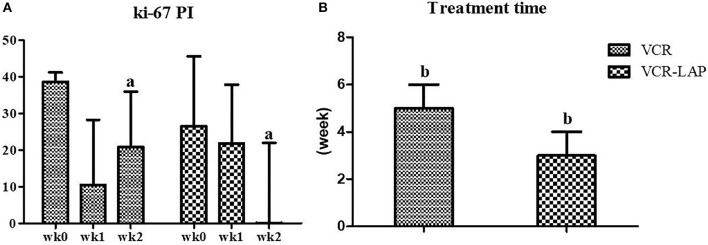
Ki-67 proliferative index (PI) on CTVT sample before treatment (wk0) and during treatment (wk1–2). Ki-67 PI of conventional protocol revealed variable values, on the other hand, modified combination protocol shown the decreasing trend. Moreover, Ki-67 PI of modified combination protocol in wk-2 PT was lower than the conventional protocol significantly (a = 0.024) **(A)**. The treatment time of modified combination protocol was shorter than conventional protocol significantly (b = 0.019) **(B)** (Mann-Whitney *U*-test was used to determine the significance of differentiation between the conventional and modified combination chemotherapeutic protocols, ^a, b^*p* < 0.05).

Nine dogs from 11 cases that were treated with VCR (81.82%) showed complete remission within the average of 5 weeks of treatment duration. There were 2 ETVT cases that developed vincristine resistance. In the first case, there was a large oro-nasal mass. For this case, combination chemotherapy was applied using simultaneous vincristine (IV) and L-asparaginase (SC), along with the vincristine resistance protocol every 2 weeks, and the dog showed complete remission in 3 applications. The second case had a preputial mass with inguinal lymph node involvement. Twelve dogs from 13 cases (92.31%) showed complete remission within an average of 3 weeks of treatment duration, with VCR-LAP ranging from 2 to 5 weeks. The treatment time of VCR-LAP was significantly shorter than VCR (Figure 2B). In addition, one dog (No. 8, Table 5) died during the first 2 weeks of treatment with the normal blood profile. However, necropsy was not performed upon the owner's refusal. This dog was included in clinical-pathological study while it was not included for response to therapy evaluation.

According to the VCOG-CTCAE grading, the weight loss and gastrointestinal toxicity (anorexia, nausea and vomiting) were the two main common side effects. Moreover, lethargy grade 1 was observed in one dog (1/11, 9.09%) and two dogs (2/13, 15.38%) in VCR and VCR-LAP, respectively. The weight loss grade 1 (5/11, 45.45%) and grade 2 (1/11, 9.09%) were recorded in VCR. In addition, the weight loss grade 1 and grade 3 were found in VCR-LAP. For the gastrointestinal toxicity, anorexia grade 1 (3/11, 27.27%) and grade 2 (2/11, 18.18%) were observed in VCR. As anorexia grade 1 (3/13, 23.08%) and grade 2 (1/13, 7.69%) were observed in VCR-LAP, respectively (Tables 4, 5). Leukopenia was observed but it was still observed in grade 0 of hematological toxicity grade in both protocols (the data did not show).

### Tumor-Infiltrating Lymphocytes and Fibrosis in Tumor Regression After Chemotherapeutic Treatment

**Figure 3:**
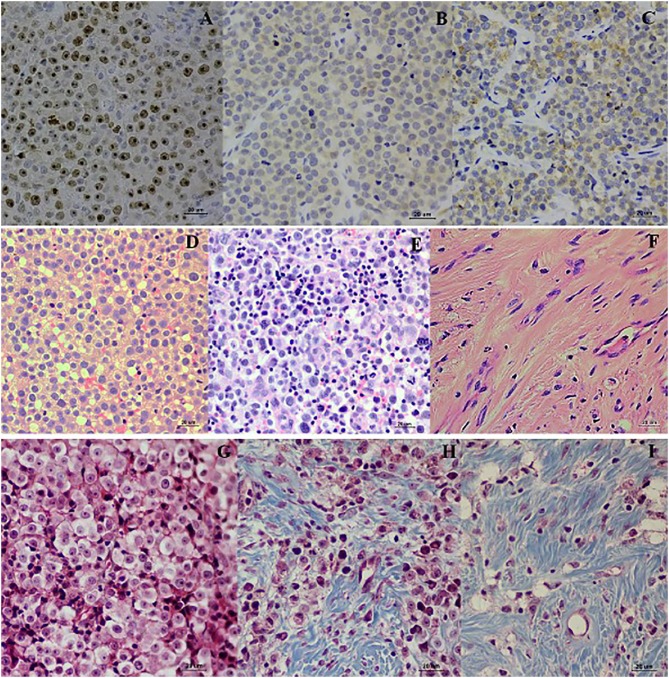
Localization of Ki-67, Bcl-2, and Bax. Ki-67 localized in the nucleus of TVT cells **(A)**. Bcl-2 localized in nuclear membrane and cytoplasm of CTVT cells **(B)**. Bax localized in the cytoplasm of CTVT cells **(C)** (LSAB-IHC method, DAB counterstained with Mayer, hematoxylin, bar = 20 μm). Histopathology pictures of CTVT in progression phase and regression phase. CTVT during growth phase **(D,G)**, the section shown the numerous CTVT cells. CTVT during early regression phase **(E,H)**, the section showed the moderate amount of CTVTs and presented the tumor infiltrated lymphocytes (TILs). CTVT during the late stage of regression phase **(F,I)**, fibrous tissue which replaced the CTVT parenchyma (H&E stain, bar = 20 μm) (Masson Trichrome stain, bar = 20 μm).

The wk0 tissue section showed numerous round to ovoid tumor cells arranged in solid sheets. The tumor cells had round to ovoid nuclei with prominent nucleoli and coarse chromatin. They had pale vacuolated cytoplasm. Few mitotic figures were also found (Figures 3D,G). After chemotherapeutic treatment, the lymphocytes gradually increased and, at the same time, the population of CTVT cells decreased (Figure 3E). At the end of the treatment, the CTVT cells were replaced by fibrous tissue (Figures 3F,H,I). The fibrosis area of wk0, wk1-PT, and wk2-PT which presented in the blue color from Masson's Trichrome Staining, were also increasing significantly when compared before and after treatment (Figure 1B). Though, there was no statistical significance among treatment protocols.

The data of wk0, wk1-PT and wk2-PT showed that TILs/CTVT ratio of the treated groups significantly increased when compared to the pre-treatment group within the same chemotherapeutic protocols (Figure 1C and Supplement data Table 1). However, there was no statistical significance among treatment protocols.

To evaluate the proliferation of CTVT cells, Ki-67 proliferative index (Ki-67 PI) was performed by IHC (Figure 3A). Ki-67 PI tended to decrease in the VCR-LAP, Ki-67 PI was 26.470 (0–66.470) in wk0 and decreased to 21.78 (0–69.030) and 0 (0–48.540) in wk1 and wk2-PT, but this event was not found in the VCR. Moreover, Ki-67 PI in VCR showed no difference between the pre-treated and treated groups (Supplement data Table 1). Interestingly, Ki-67 PI of the VCR-LAP was significantly lower than VCR in wk2-PT (*p* = 0.024) (Figure 2A).

### Apoptosis Related to Tumor Regression as a Result of Chemotherapeutic Treatment

**Figure 4:**
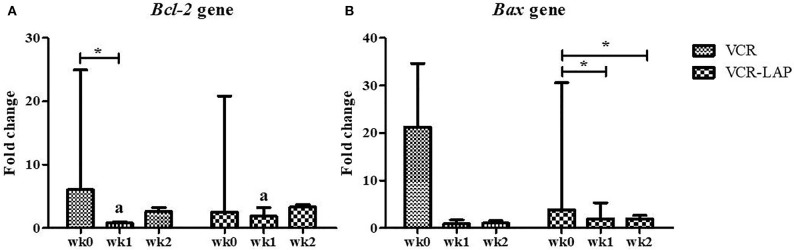
Relative mRNA expression of *Bcl-2* **(A)** and *Bax* **(B)** in CTVT dogs before treatment (wk0) and during treatment (wk1–2 PT). Relative mRNA expression of *Bcl-2* in wk1 lower than wk0 in CTVT dogs treated with VCR. In addition, relative mRNA expression of *Bax* during treatment lower than wk0 in CTVT dogs treated with VCR-LAP [Friedman repeated measures analysis of variance and Wilcox Signed-Rank test was used to determine the significance of differentiation before (wk0) and during treatment (wk1 and wk2) in each group, ^*^p < 0.05]. Relative mRNA expression of *Bcl-2* in CTVT dogs treated with VCR lower than VCR-LAP in wk1 significantly (Mann-Whitney *U*-test was used to determine the significance of differentiation between the conventional and modified combination chemotherapeutic protocols, ^a^*p* < 0.05).

The relative mRNA expression of *Bcl-2* and *Bax* in both chemotherapeutic groups had a trend to decrease from pre-treatment. *Bcl-2* expression was significantly decreased after treatment (wk1-PT) in VCR (*p* = 0.006, Supplement data Table 2). In addition, *Bax* expression significantly decreased in the VCR-LAP at wk1 (*p* = 0.019) and wk2-PT (*p* = 0.023) (Figure 4B and Supplement data Table 2). In VCR-LAP, *Bcl-2* expression of wk1-PT was higher than VCR (*p* = 0.027, Figure 4A).

**Figure 5:**
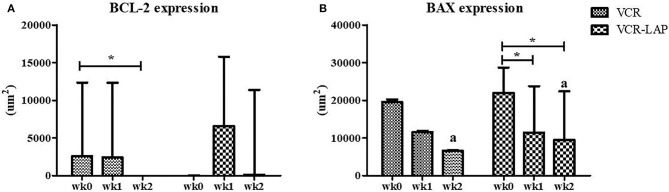
Expression of the apoptotic protein Bcl-2 **(A)** and Bax **(B)** in CTVT dogs before treatment (wk0) and during treatment (wk1–2 PT). BCL-2 expression (VCR) in wk 2 lower than wk0 significantly. Moreover, BAX expression (VCR-LAP) after treatment lower than before treatment significantly [Friedman repeated measures analysis of variance and Wilcox Signed-Rank test was used to determine the significance of differentiation before (wk0) and during treatment (wk1 and wk2) in each group, ^*^p < 0.05]. BAX protein expression of the VCR-LAP protocol was significantly higher than the VCR protocol at wk2-PT (Mann-Whitney *U*-test was used to determine the significance of differentiation between the conventional and modified combination chemotherapeutic protocols, ^a^*p* < 0.05).

BCL-2 and BAX protein expression of samples were observed and measured by average positive immunolabeling area (Figures 3B,C). The result of wk1-PT and wk2-PT also showed a decreasing trend similar to the relative mRNA expression. BCL-2 of wk2-PT significantly decreased from wk0 (*p* = 0.025, Figure 5A and Supplement data Table 2) in VCR. In the same way, BAX expression of VCR-LAP in wk1 and wk2-PT were significantly lower than the pre-treatment group (*p* = 0.019, *p* = 0.019, Figure 5B and Supplement data Table 2). Interestingly, BAX expression of VCR-LAP was significantly higher than VCR at wk2-PT (*p* = 0.046, Figure 5B). Moreover, the BAX/BCL-2 ratio between two chemotherapeutic protocols had no significant difference.

### ABCB1 and ABCG2 Gene Expression Profile During Pre-treatment and Post-treatment

**Figure 6:**
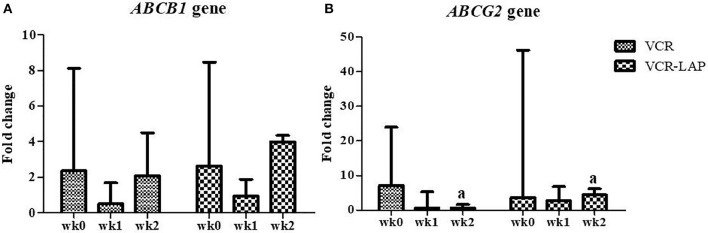
Relative mRNA expression of the ABC transporters *ABCB1* **(A)** and *ABCG2* **(B)** in CTVT dogs before treatment (wk0) and during treatment (wk1–2 PT). Relative mRNA expression of *ABCG2* of CTVT dogs treated with VCR lower than VCR-LAP in wk2 significantly (Mann-Whitney *U*-test was used to determine the significance of differentiation between the conventional and modified combination chemotherapeutic protocols, ^a^*p* < 0.05).

The relative mRNA expression of the *ABCB1* gene in both chemotherapeutic protocols showed a decreasing trend in wk1-PT and then increased in wk2-PT, but the relative expressions were not more than wk0. However, there was no significant difference between those chemotherapeutic groups (Figure 6A and Supplement data Table 3). In addition, the relative mRNA expression of *ABCG2* gene in both chemotherapeutic groups tended to decrease from wk0 (Figure 6B and Supplement data Table 3). Surprisingly, *ABCG2* expression of VCR-LAP was significantly higher than VCR (*p* = 0.035, Figure 6B). However, the relative mRNA expression of the sample before the treatment was higher than after treatment.

## Discussion

Canine transmissible venereal tumor is found in the tropical and subtropical climate region, especially in a poorly controlled, free-roaming dog population (13). Therefore, owned free-ranging dogs and stray dogs are the main populations of CTVT dogs and it is more common in intact adult dogs as in this study (15, 35). Interestingly, from or results, we found that dogs of an older age usually develop ETVT rather than GTVT, which is more common in young adults. The poor immune status and body condition might both be cofactors in ETVT aging dogs. CTVT usually occurred in the genital area with a low metastatic rate which <20% (15). However, it can be found in the extragenital area (4–7). Moreover, the skin/subcutis was the main site followed by inguinal lymph nodes (4, 7, 42).

VCR-LAP protocol showed an effective result and gave a shorter treatment time than VCR. Moreover, the average cost of combination protocol (3 weeks, 260–280 USD) was also less than the conventional protocol (5 weeks, 315–330 USD). Normally, LAP has been used for short induction and rarely used for maintenance to avoid resistance and serious toxicity (20, 21, 43, 44). It is active in the G1 phase of the cell cycle, which is an important point that differs in normal cells and tumor cells (45). Since LAP can cause hypersensitivity and pancreatitis in dogs (43–45), we applied LAP in a lower dose than in the previous reports and altered the route of administration from intravenously to subcutaneously to avoid adverse effects (16), which gave a satisfied outcome of complete regression. However, there was a dog that died during treatment. This finding was still controversial because the owner did not permit to make a necropsy.

In this study, two mixed cell type ETVT cases showed the resistance behavior. It can infer that not only plasmacytic type, but also mixed cell type was related to the malignant behavior. Firstly, a large oro-nasal mass, measuring more than 20 cm in diameter, was treated with more than 6 applications of VCR but it was still visible in the nasal cavity by radiography. Alternative treatments for resistant cases in previous reports were mono-chemotherapy by using doxorubicin (15), or combination chemotherapy by using a combination of vincristine and L-asparaginase (VCR-LAP) (16), or surgery (13, 46), and/or radiation therapy (13, 15). The second case had a chronic bite, which was sustained at the prepuce and had inguinal lymph node enlargement. After being diagnosed this dog received 6 applications of VCR with complete response. During the post treatment monitoring period, he was accidentally involved in a dog fight, and then enucleation was performed. After enucleation, CTVT was diagnosed from the ocular mass. Therefore, this point was noted and recommended for a long-term monitoring period (more than 6 months) after the final chemotherapeutic application (13, 15, 46).

TILs/CTVT ratio of the treated groups increased significantly when compared with the pre-treatment groups in the same chemotherapeutic protocols. TILs might accompany the chemotherapy causing tumor apoptosis and finally result in tumor regression. Normally, only lymphocytes that secrete interleukin-6 (IL-6) play an important role on the cells and cytokines by acting against TGF-β1 secreted from CTVT cells, leading to spontaneous regression (33). This could be implied that chemotherapy and TILs provide a synergistic effect during treatment. In addition, there was a significant increase in the amount of fibrosis in the treated samples compared with the pre-treated samples (1, 11, 12). The tumor evades the normal tissue due to tissue injury, so the progression of tumor cells ceased, and the tumor parenchyma collapsed. As the healing process of the host starts, there is an increase of fibrosis and collagen formation.

Ki-67 PI is related to tumor progression, metastatic ability, and prognostic value (30, 31, 36). It was higher in the pre-treated samples than the post-treated samples. The cellular proliferation activity decreased during the chemotherapy-induced regression period. According to this study, Ki-67 PI of wk2-PT in VCR-LAP was lower than VCR. High Ki-67 PI indicated high proliferation ability of the tumor cells. The VCR protocol had a higher growth fraction of CTVT cells than the VCR-LAP protocol. Therefore, the CTVT proliferation activity was decreased by VCR-LAP rather than VCR.

Bcl-2 overexpression indicates a malignant feature (29) and causes chemotherapeutic drug resistance with associated high mortality rate in previous reports (28). On the other hand, decrease or loss of Bcl-2 expression related to aggressiveness in basal cell carcinoma (47), and resulting in improved patient survival in human breast carcinoma (25). Furthermore, a higher rate of apoptosis was related to the less aggressive behavior in CTVT cases (48). According to this information, it may imply that Bcl-2 overexpression is related to tumor progression and tumor survival factor in tumor cells (49). However, Bcl-2 overexpression was independent of the development stage of CTVT cells (50, 51). We also found that VCR and LAP induced apoptosis were accompanied by down regulation of *Bcl-2* and *Bax* mRNA expression. The low or decrease in Bcl-2 expression is associated with treatment response in human breast cancer studies (26, 27), similar to the lack or low Bax expression from 5-FU-based adjuvant therapies in human colorectal carcinoma (52). Our results showed that *Bax* and *Bcl-2* mRNA expression and BAX and BCL-2 protein expression did not differ between treatment protocols and time of treatment. These results might be the cause of P53 activity in CTVT cells due to the limitation of Bax production. In addition, *Bcl-2* expressions may be down-regulated by P53 through binding to the silencer of Bcl-2, and p53 plays the important role in apoptosis after treatment. Moreover, when comparing the Bax/Bcl-2 ratio, there was no significant difference between two chemotherapeutic protocols. High BCL-2 expression was found only in the pre-treated samples from both protocols and the expression was also higher than the normal tissue from the penis. This may confirm that Bcl-2 is necessary for the CTVT progression phase and survival ability as previously mentioned (49). This result may imply that both low expression of Bcl-2 and Bax after chemotherapeutic treatment correlated with good prognostic outcome. Moreover, the resistant cases in this study did not show as Bcl-2 overexpression.

*ABCB1* gene in both protocols showed a decreasing trend in wk1-PT and then increased in wk2-PT, but they were not more than those of the pre-treated samples. However, there was no significant difference between the chemotherapeutic protocol groups (Supplement data Table 3). According to previous studies, vincristine resistance is related to the increase of P-glycoprotein expression. Given these findings, *ABCB1* expression was also directly related to chemotherapeutic response. As mentioned, P-glycoprotein pumps the cytotoxic agents outside of the cells, which reduce the cytotoxic lethal concentration. However, a lower *ABCB1* expression has been reported, meaning this situation is still under controversy. The low expression of *ABCB1* after treatment may require a higher level of chemotherapeutic induction (48). From CTVT studies, *ABCB1, MRP1*, and *MRP2* have been studied, but there is no data on *ABCG2* expression. Our results showed that relative mRNA expression of the *ABCG2* gene in both chemotherapeutic groups had a decreasing trend (Supplement data Table 3). It might be stated that *ABCG2*, which is normally related to the doxorubicin-resistant situation, may not be related to VCR-LAP resistant phenomena in this study. Therefore, this supports our results that the dose of chemotherapeutic protocols was appropriate, in order to reduce the occurrence of resistance phenomena. However, there were some cases that showed vincristine resistance, but did not show a high expression of *ABCB1* and *ABCG2*, so further studies are necessary to clarify these aspects. This may be due variability of the gene and transcribed protein in an *in vivo* study.

In conclusion, VCR- LAP, proposed in this study, might be a new choice of chemotherapy in CTVT cases, and also for treating resistant VCR-treated CTVT cases. Furthermore, this protocol also provides a shorter period of treatment. This advantage could lower the chance of the resistance phenomenon. Moreover, this study also revealed more information about *Bcl-2 and Bax, ABCB1*, and *ABCG2* mRNA expression and Bcl-2 and Bax expression *in vivo*. However, there was a limited sample size and also a short duration of follow up after treatment. For further studies, more CTVT sample cases should be obtained and a longer duration of follow up after treatment should be done.

## Data Availability

All datasets generated for this study are included in the manuscript/Supplementary Files.

## Ethics Statement

The animal study was reviewed and approved by Chulalongkorn University Animal Care and Use Committee. Written informed consent was obtained from the owners for the participation of their animals in this study.

## Author Contributions

CS designed the experiments, collected the samples, organized the experiments, analyzed the data, and drafted the manuscript. PT collected the samples and organized the experiments. STa analyzed the data and revised the manuscript draft. STe designed the experiment and revised the manuscript draft. AR designed the experiments and revised the manuscript draft.

### Conflict of Interest Statement

The authors declare that the research was conducted in the absence of any commercial or financial relationships that could be construed as a potential conflict of interest.

## Supplementary Material

Supplement data Figure 1 Pre-treatment and post-treatment of CTVT cases. The ETVT masses were involved with the ocular area, oral mucosa and subcutaneous area (**A**, Pre-treatment; **B**, Post-treatment). The cauliflower-like mass lesion located at the external genital area, GTVT, of male and female dog (**C**, **E**, Pre-treatment; **D,F**, Post-treatment). The button-like mass feature was found in the subcutaneous area and the large mass invaded the normal structure of hindlimb (**G**, Pre-treatment; **H**, Post-treatment).

Supplement data Table 1 Tumor relative volume, TIL/CTVT ratio, the percentage of Ki-67 positive cells before treatment, after 1 and 2 weeks of treatment (median, interquartile range) (Friedman repeated measures analysis of variance and Wilcoxon Signed-Rank Test, ^*^*p* < 0.05).

Supplement data Table 2 BCL-2 and BAX IHC positive area and relative mRNA expression of *Bcl-2* and *Bax* gene (median, interquartile range) (Friedman repeated measures analysis of variance and Wilcoxon Signed-Rank Test, ^*^*p* < 0.05).

Supplement data Table 3 Relative mRNA expression of *ABCB1* and *ABCG2* gene (median, interquartile range) (Friedman repeated measures analysis of variance and Wilcoxon Signed-Rank Test, ^*^*p* < 0.05).

Supplement data Table 4 Ranking of reference genes in canine transmissible venereal tumor from high to low stability by various algorithms. Stability values are in brackets.
