# Supplementary figures and images for: Conventional-Vincristine Sulfate vs. Modified Protocol of Vincristine Sulfate and L-Asparaginase in Canine Transmissible Venereal Tumor
Source: Front Vet Sci. 2019 Sep 18;6:300. doi: 10.3389/fvets.2019.00300 (PMC6759545; doi:10.3389/fvets.2019.00300)

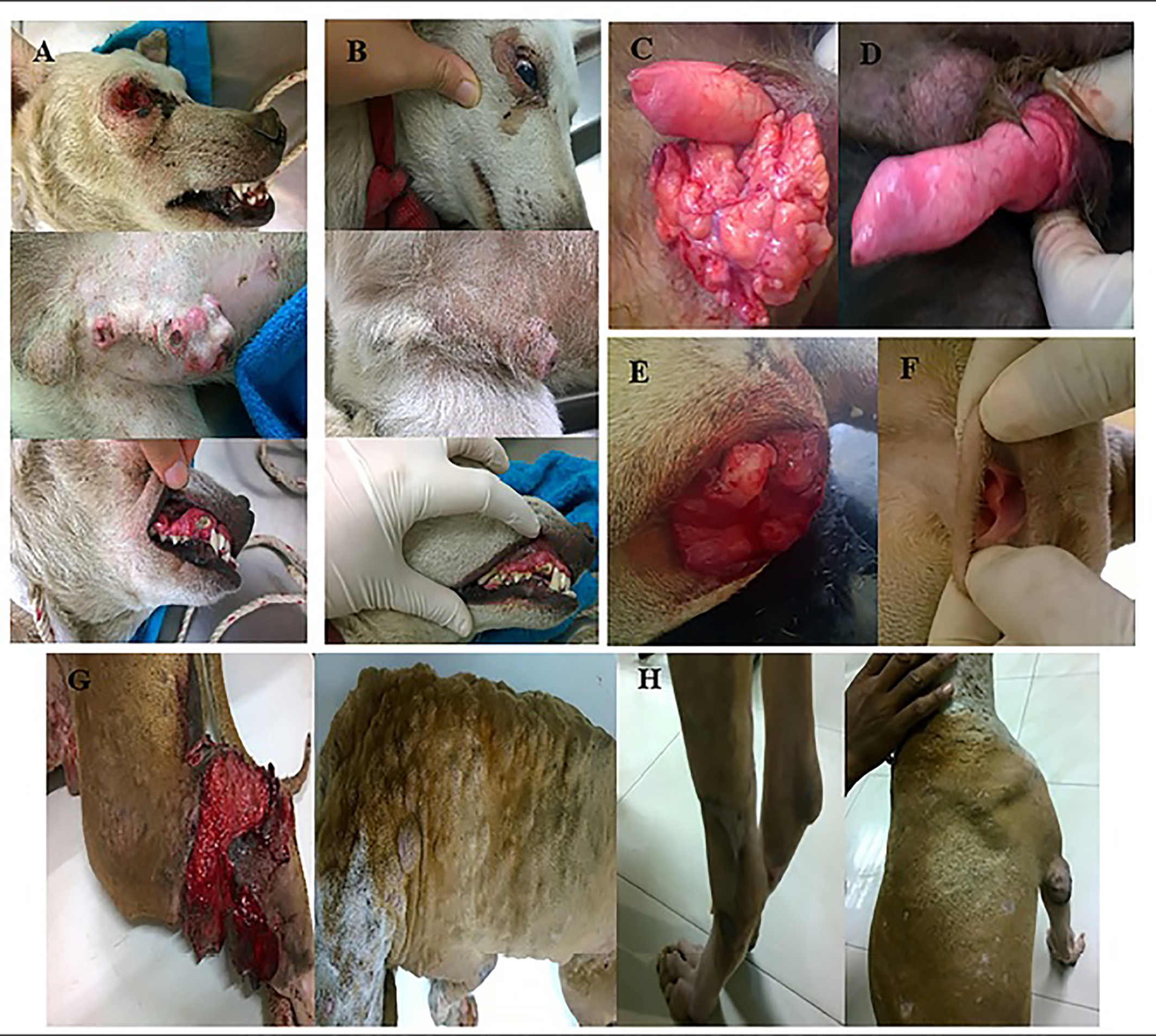

Supplement: Supplement data Figure 1 — Pre-treatment and post-treatment of CTVT cases. The ETVT masses were involved with the ocular area, oral mucosa and subcutaneous area (A, Pre-treatment; B, Post-treatment). The cauliflower-like mass lesion located at the external genital area, GTVT, of male and female dog (C, E, Pre-treatment; D,F, Post-treatment). The button-like mass feature was found in the subcutaneous area and the large mass invaded the normal structure of hindlimb (G, Pre-treatment; H, Post-treatment). [file Image_1.TIF]
