# Supplementary material for: Conventional-Vincristine Sulfate vs. Modified Protocol of Vincristine Sulfate and L-Asparaginase in Canine Transmissible Venereal Tumor
Source: Front Vet Sci. 2019 Sep 18;6:300. doi: 10.3389/fvets.2019.00300 (PMC6759545; doi:10.3389/fvets.2019.00300)
Supplement: Supplement data Table 1 — Tumor relative volume, TIL/CTVT ratio, the percentage of Ki-67 positive cells before treatment, after 1 and 2 weeks of treatment (median, interquartile range) (Friedman repeated measures analysis of variance and Wilcoxon Signed-Rank Test, *p < 0.05). [file Table_1.DOCX]

**Supplement data Table 1** Tumor relative volume, TIL/CTVT ratio, the percentage of Ki-67 positive cells before treatment, after 1 and 2 weeks of treatment (median, interquartile range) (Friedman repeated measures analysis of variance and Wilcoxon Signed-Rank Test, * *p*<0.05)

| **Treatment** | **Parameter** | **Wk0** | **Wk1-PT** | ***p*-value** | **Wk2-PT** | ***p*-value** |
| --- | --- | --- | --- | --- | --- | --- |
| Conventional | Tumor relative volume | 0.097 | 0.015* | 0.003 | 0.001* | 0.003 |
| (VCR) |  | (0.045-0.250) | (0.002-0.022) |  | (0-0.008) |  |
|  | TIL/CTVT ratio | 0.070 | 0.510^*^ | 0.006 | 3.280* | 0.016 |
|  |  | (0.020-0.110) | (0.340-2.530) |  | (0-4.290) |  |
|  | Fibrosis area (µm^2^) | 6.259x10^3^ | 6.665x10^3^ | 0.286 | 35.606x10^3^ | 0.028 |
|  |  | (6.109-7.485x10^3^) | (5.534-38.505x10^3^) |  | (35.498-43.007x10^3^) |  |
|  | Ki-67 (%) | 38.630 | 10.53 | 0.630 | 20.83 | 0.630 |
|  |  | (0-41.230) | (0-28.310) |  | (16.670-35.960) |  |
| **Treatment** | **Parameter** | **Wk0** | **Wk1-PT** | ***p*-value** | **Wk2-PT** | ***p*-value** |
| Modified | Tumor relative volume | 0.100 | 0.016* | 0.001 | 0.001* | 0.001 |
| combination |  | (0.049-0.255) | (0.004-0.112 |  | (0-0.014) |  |
| (VCR-LAP) | TIL/CTVT ratio | 0.150 | 0.960* | 0.001 | 3.30* | 0.013 |
|  |  | (0.110-0.215) | (0.555-1.810) |  | (0-9.195) |  |
|  | Fibrosis area (µm^2^) | 7.116x10^3^ | 33.297x10^3^* | 0.006 | 34.214x10^3*^ | 0.007 |
|  |  | (6.015-7.832x10^3^) | (23.258-35.790x10^3^) |  | (29.754-39.535x10^3^) |  |
|  | Ki-67 (%) | 26.470 | 21.780 | 0.115 | 0 | 0.115 |
|  |  | (0-45.595) | (11.295-37.835) |  | (0-22.035) |  |
