# Supplementary material for: Conventional-Vincristine Sulfate vs. Modified Protocol of Vincristine Sulfate and L-Asparaginase in Canine Transmissible Venereal Tumor
Source: Front Vet Sci. 2019 Sep 18;6:300. doi: 10.3389/fvets.2019.00300 (PMC6759545; doi:10.3389/fvets.2019.00300)
Supplement: Supplement data Table 2 — BCL-2 and BAX IHC positive area and relative mRNA expression of Bcl-2 and Bax gene (median, interquartile range) (Friedman repeated measures analysis of variance and Wilcoxon Signed-Rank Test, *p < 0.05). [file Table_2.DOCX]

**Supplement data Table 2** BCL-2 and BAX IHC positive area and relative mRNA expression of *Bcl-2* and *Bax* gene (median, interquartile range) (Friedman repeated measures analysis of variance and Wilcoxon Signed-Rank Test, * *p*<0.05)

| **Treatment** | **Parameter** | **Wk0** | **Wk1-PT** | ***p*-value** | **Wk2-PT** | ***p*-value** |
| --- | --- | --- | --- | --- | --- | --- |
| Conventional | BCL-2 (µm^2^) | 2.563x10^3^ | 2.414x10^3^ | 0.575 | 0x10^3^* | 0.025 |
| (VCR) |  | (0-12.358x10^3^) | (0-12.332x10^3^) |  | (0-0) |  |
|  | BAX (µm^2^) | 19.563x10^3^ | 11.559x10^3^ | 0.135 | 6.591x10^3^ | 0.135 |
|  |  | (14.183x10^3^-20.214x10^3^) | (5.448x10^3^-11.890x10^3^) |  | (4.851x10^3^-6.712x10^3^) |  |
|  |  |  |  |  |  |  |
|  | *Bcl-2* | 6.060 | 0.800* | 0.006 | 2.620 | 0.063 |
|  |  | (1.590-24.930) | (0.350-1.010) |  | (0.129-3.230) |  |
|  | *Bax* | 21.260 | 0.950 | 0.180 | 1.050 | 0.180 |
|  |  | (1.000-34.660) | (0.550-1.730) |  | (0.900-1.590) |  |
| **Treatment** | **Parameter** | **Wk0** | **Wk1-PT** | ***p*-value** | **Wk2-PT** | ***p*-value** |
| Modified | BCL-2 (µm^2^) | 0x10^3^ | 6.581x10^3^ | 0.169 | 0.133x10^3^ | 0.169 |
| Combination |  | (0-17.543x10^3^) | (0.117-15.780x10^3^) |  | (0-11.374x10^3^) |  |
| (VCR-LAP) |  |  |  |  |  |  |
|  | BAX (µm^2^) | 21.988x10^3^ | 11.434x10^3^* | 0.019 | 9.477x10^3^* | 0.019 |
|  |  | (14.508x10^3^-28.709x10^3^) | (8.201-23.736x10^3^) |  | (0-22.390x10^3^) |  |
|  |  |  |  |  |  |  |
|  | *Bcl-2* | 2.550 | 1.890 | 0.695 | 3.320 | 0.695 |
|  |  | (0.540-20.820) | (0.840-3.230) |  | (1.360-3.719) |  |
|  |  |  |  |  |  |  |
|  | *Bax* | 3.860 | 1.935* | 0.019 | 1.975* | 0.023 |
|  |  | (1.575-30.595) | (0.918-5.325) |  | (0.873-2.663) |  |
