# Supplementary material for: Conventional-Vincristine Sulfate vs. Modified Protocol of Vincristine Sulfate and L-Asparaginase in Canine Transmissible Venereal Tumor
Source: Front Vet Sci. 2019 Sep 18;6:300. doi: 10.3389/fvets.2019.00300 (PMC6759545; doi:10.3389/fvets.2019.00300)
Supplement: Supplement data Table 3 — Relative mRNA expression of ABCB1 and ABCG2 gene (median, interquartile range) (Friedman repeated measures analysis of variance and Wilcoxon Signed-Rank Test, *p < 0.05). [file Table_3.DOCX]

**Supplement data Table 3** Relative mRNA expression of *ABCB1* and *ABCG2* gene (median, interquartile range)

(Friedman repeated measures analysis of variance and Wilcoxon Signed-Rank Test, * *p*<0.05)

| **Treatment** | **Parameter** | **Wk0** | **Wk1-PT** | ***p*-value** | **Wk2-PT** | ***p*-value** |
| --- | --- | --- | --- | --- | --- | --- |
| Conventional | *ABCB1* gene | 2.360 | 0.510 | 0.115 | 2.08 | 0.115 |
| (VCR) |  | (0.535-8.113) | (0.390-1.685) |  | (0.750-4.498) |  |
|  | *ABCG2* gene | 7.140 | 0.700 |  | 0.670 |  |
|  |  | (0.260-23.920) | (0.190-5.310) |  | (0.460-1.610) |  |
| **Treatment** | **Parameter** | **Wk0** | **Wk1-PT** |  | **Wk2-PT** |  |
| Modified | *ABCB1* gene | 2.640 | 0.940 | 0.178 | 3.993 | 0.178 |
| combination |  | (0.510-8.460) | (0.310-1.890) |  | (1.490-4.350) |  |
| (VCR-LAP) | *ABCG2* gene | 3.730 | 2.790 | 0.307 | 4.426 | 0.307 |
|  |  | (0.880-46.210) | (0.810-6.770) |  | (1.790-6.150) |  |
