# Supplementary material for: Conventional-Vincristine Sulfate vs. Modified Protocol of Vincristine Sulfate and L-Asparaginase in Canine Transmissible Venereal Tumor
Source: Front Vet Sci. 2019 Sep 18;6:300. doi: 10.3389/fvets.2019.00300 (PMC6759545; doi:10.3389/fvets.2019.00300)
Supplement: Supplement data Table 4 — Ranking of reference genes in canine transmissible venereal tumor from high to low stability by various algorithms. Stability values are in brackets. [file Table_4.DOCX]

**Supplement data Table 4** Ranking of reference genes in canine transmissible venereal tumor from high to low stability by various algorithms. Stability values are in brackets.

| **Rank** | **RefFinder** | **Normfinder** | **Bestkeeper** | **Delta CT** | **Genorm** |
| --- | --- | --- | --- | --- | --- |
| 1 | *Β*-actin^a^  (1.41) | *Β*-actin  (0.47) | *Β*-actin  (1.10) | *Β*-actin  (2.69) | *Β*-actin  (0.95) |
| 2 | HPRT^b^  (3.00) | HPRT  (1.87) | HPRT  (1.24) | HPRT  (3.28) | HPRT  (1.55) |
| 3 | TBP^c^  (4.00) | TBP  (2.78) | TBP  (2.03) | TBP  (3.61) | TBP  (2.00) |
| 4 | GAPDH^d^  (5.00) | GAPDH  (6.08) | GAPDH  (4.38) | GAPDH  (6.24) | GAPDH  (3.70) |

^a^*Β*-actin (beta-actin) (Pisamai *et al.*, 2016)

^b^HPRT (hypoxanthine phosphoribosyltransferase) (Culmsee *et al*., 2004)

^c^TBP (TATA box binding protein) (Peters *et al.*, 2007)

^d^GAPDH (glyceraldehydes-3-phosphate gehydrogenase) (Pisamai *et al.*, 2016)
